# Unusual Radiological Presentation of a Typical Carcinoid in a Young Female With High-Grade Intermittent Fever: A Case Report

**DOI:** 10.7759/cureus.66777

**Published:** 2024-08-13

**Authors:** Karpagam Kannadasan, Sakthi Ganesh Subramonian, Yuvaraj Muralidharan, Karthik Krishna Ramakrishnan, Paarthipan Natarajan

**Affiliations:** 1 Department of Radiology, Saveetha Medical College and Hospital, Saveetha Institute of Medical and Technical Sciences (SIMATS), Saveetha University, Chennai, IND

**Keywords:** typical variant, diagnostic challenges, high-grade fever, neuroendocrine tumor, pulmonary carcinoid

## Abstract

Pulmonary carcinoids, particularly the typical variant, are low-grade neuroendocrine tumors (NETs) known for their often indolent behavior. Their rare and atypical presentations can pose significant diagnostic challenges. We present a case of a 39-year-old female with no significant past medical history who presented with high-grade intermittent fever, chills, rigors, and breathlessness, with symptoms persisting for approximately 20 days. Initial investigations, including chest X-ray and contrast-enhanced computed tomography scans, revealed a homogenously enhancing lesion in the left lower lobe, raising concerns of a malignant process. A percutaneous lung biopsy was performed to further evaluate the lesion. Histopathological examination confirmed a diagnosis of Grade 1 NET (typical carcinoid), supported by elevated levels of Synaptophysin and Chromogranin A on immunohistochemistry. This case highlights the varied clinical presentations of pulmonary carcinoid tumors and underscores the importance of comprehensive radiological and histopathological assessments for accurate diagnosis and effective management.

## Introduction

Broncho-pulmonary carcinoids, representing 1-2% of all lung malignancies, constitute a rare subset of neuroendocrine tumors (NETs) originating from the bronchial epithelium. These neoplasms are categorized into two primary types: typical carcinoids, generally low-grade with a more favorable prognosis, and atypical carcinoids, which exhibit a more aggressive phenotype characterized by elevated mitotic activity and occasional necrosis [1,2].

The clinical manifestations of pulmonary carcinoids can range from asymptomatic cases to symptomatic presentations, including cough, hemoptysis, and, less frequently, systemic symptoms such as fever of unknown origin. In addition to these general symptoms, it is important to note that bronchopulmonary carcinoids may present with specific biochemical features. Elevated levels of NET markers, such as chromogranin A (CgA), are often observed in these cases. Other markers that may be elevated include serotonin and urinary 5-HIAA (5-hydroxyindoleacetic acid) [3].

Broncho-pulmonary carcinoids also have the potential for endocrine production, leading to various paraneoplastic syndromes. For instance, bronchopulmonary malignancies can produce adrenocorticotropic hormone (ACTH), which may result in ectopic Cushing's syndrome. This syndrome is characterized by features such as central obesity, hypertension, glucose intolerance, and a characteristic "moon face." The endocrine activity of the tumor can significantly influence the clinical presentation, and thus, it is crucial to consider the potential for different manifestations based on the tumor's endocrine production.

Radiological imaging is crucial for the initial detection and evaluation of these tumors, with computed tomography (CT) scans being particularly effective in identifying features indicative of a neuroendocrine origin. Additionally, nuclear medicine plays a significant role in the diagnostic workup of pulmonary carcinoids. Imaging modalities such as 68-Gallium-DOTATATE and 68-Gallium-DOTANOC positron emission tomography (PET) scans provide valuable information by targeting somatostatin receptors, which are often overexpressed in NETs. These nuclear imaging techniques are highly sensitive and specific for detecting both primary lesions and metastatic disease, thereby enhancing the accuracy of diagnosis and staging. However, definitive diagnosis necessitates histopathological confirmation, often aided by immunohistochemical staining for neuroendocrine markers such as synaptophysin and CgA [4,5]. Histologically, typical carcinoids are distinguished by well-differentiated neuroendocrine cells, a low mitotic index, and an absence of necrosis [1,6].

Recent advancements in research have challenged conventional perceptions of pulmonary NETs, suggesting that high-grade NETs may emerge from pre-existing carcinoid tumors [7]. This evolving perspective highlights the importance of a comprehensive diagnostic strategy integrating clinical, radiological, and pathological data.

This report seeks to advance the understanding of pulmonary carcinoid tumors by emphasizing their relevance in the differential diagnosis of pulmonary lesions and systemic symptoms. By presenting this atypical case, we aim to increase awareness and advocate for a comprehensive and systematic diagnostic approach, ultimately facilitating accurate diagnoses and improving therapeutic outcomes.

## Case presentation

A 39-year-old woman with no significant prior medical history presented with a 20-day history of high-grade intermittent fever, chills, rigors, and progressive breathlessness. The patient reported that the fever was persistent, with temperatures peaking at 39.5°C (103.1°F) and fluctuating throughout the day. She experienced chills and rigors associated with the fever, which occurred intermittently. Her breathlessness had gradually worsened, making daily activities increasingly difficult. Notably, she had no history of weight loss, fatigue, or loss of appetite.

During the initial clinical evaluation, the patient’s physical examination revealed febrile episodes, with a recorded temperature of 39.2°C (102.6°F) and a mild tachycardia of 110 beats per minute. There were no significant findings on the cardiovascular or abdominal examinations. The respiratory exam noted decreased breath sounds in the left lower lung field and dullness to percussion. Despite the absence of a productive cough or hemoptysis, the patient described an increasing sense of dyspnea and discomfort in the chest.

Routine blood tests showed an elevated white blood cell count and an increased C-reactive protein level, suggesting an inflammatory or infectious process. However, no markers of autoimmune or chronic disease were identified. Given the persistence of symptoms and the lack of a clear diagnosis from initial investigations, further imaging studies were warranted.

**Figure 1:**
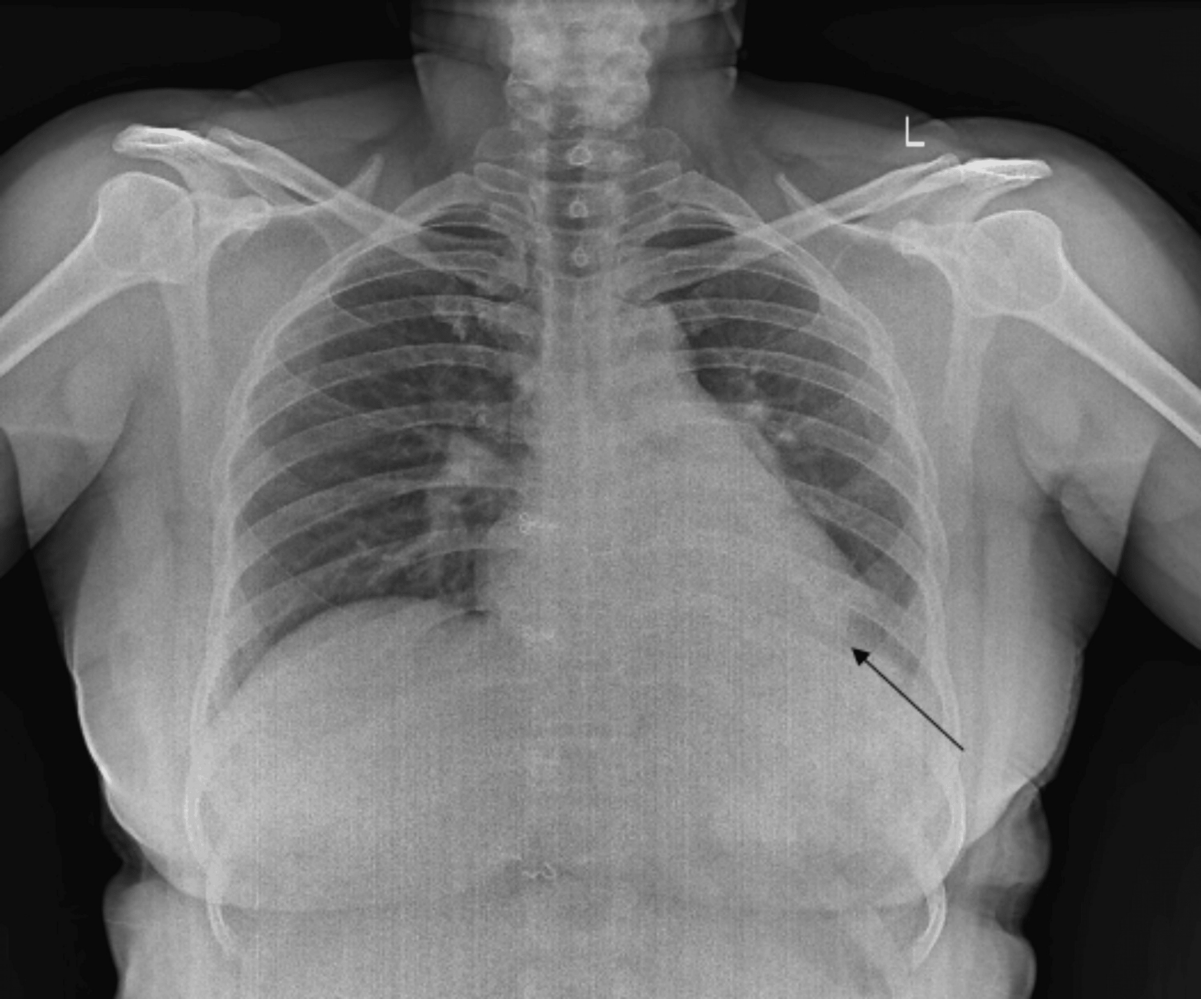
Chest radiograph reveals a suspicious collapse of the left lower lobe, indicated by a black arrow.

The initial chest radiograph (Figure 1) revealed a suspicious collapse of the left lower lobe, which was further evaluated with advanced imaging techniques.

**Figure 2:**
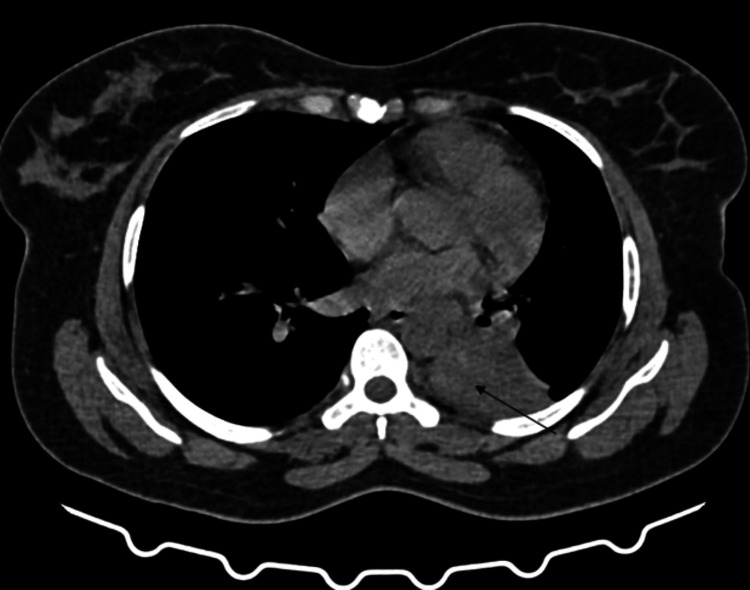
Computed tomography (CT) axial section of the thorax, using the mediastinal window, reveals a soft tissue lesion in the left lower lobe, as indicated by the black arrow.

**Figure 3:**
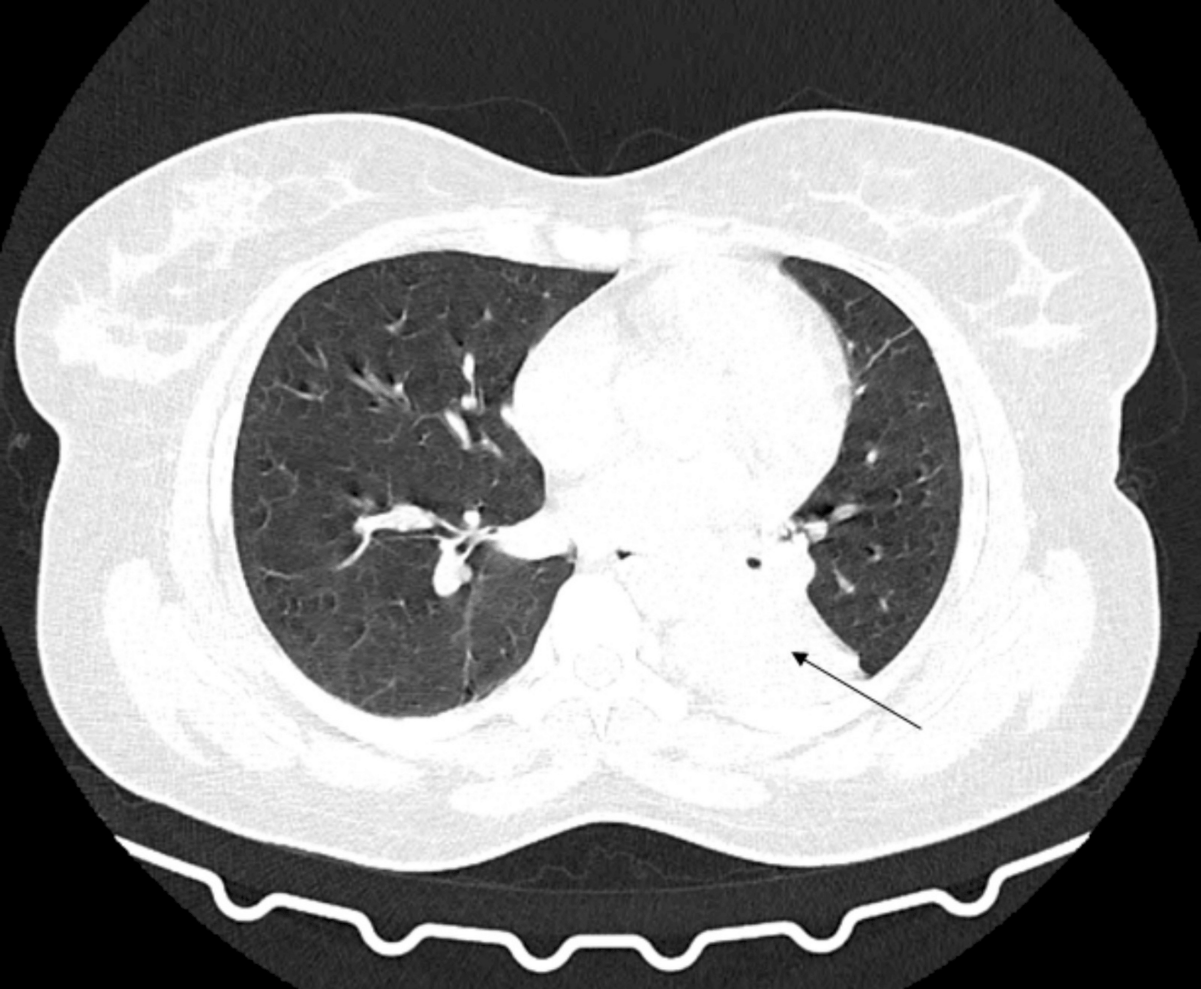
Computed tomography (CT) axial section of the thorax, using the lung window, reveals a soft tissue lesion in the left lower lobe, as indicated by the black arrow.

**Figure 4:**
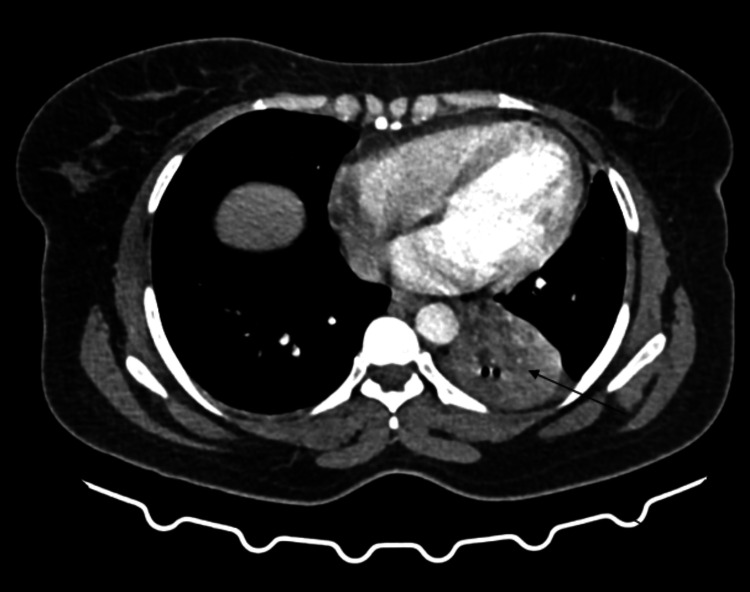
The corresponding contrast-enhanced computed tomography (CECT) thorax axial section demonstrates a fairly defined, homogeneously enhancing soft tissue lesion in the left lower lobe, accompanied by dense collapse-consolidatory changes and a few air bronchograms (black arrow).

Subsequent imaging of HRCT thorax plain and contrast is detailed in Figures 2-4. The radiographic findings demonstrated a fairly defined homogeneously enhancing lesion in the left lower lobe of the lung with associated dense collapse-consolidatory changes involving the left lower lobe, with few air bronchograms raising a high index of suspicion for malignancy. This led to a preliminary impression of a likely malignant neoplastic etiology.

**Figure 5:**
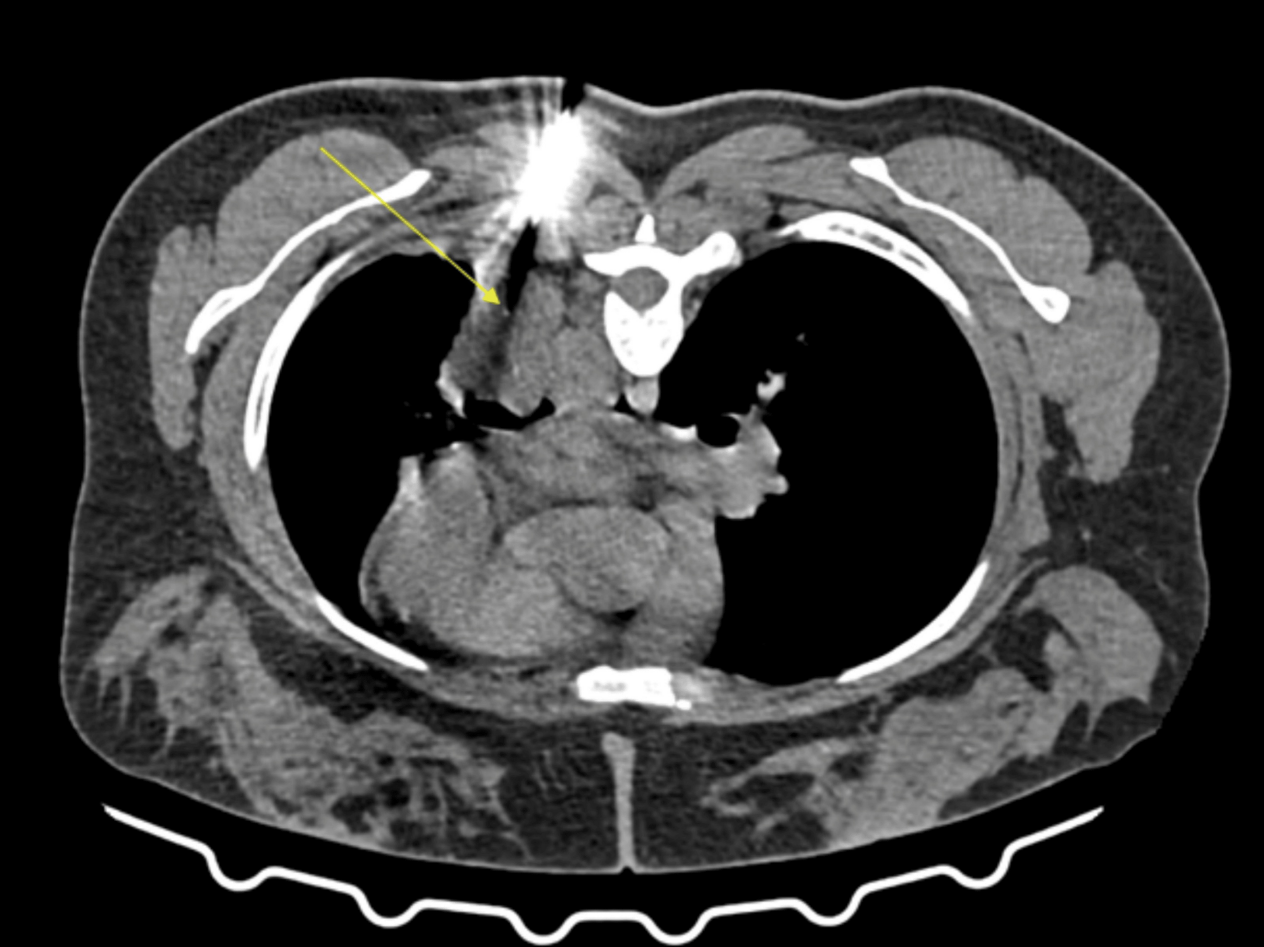
Computed tomography (CT)-guided biopsy performed in the prone position from the left lower lobe lesion shows the precise placement of the tip of the biopsy needle (yellow arrow), highlighting the technical precision.

To establish a definitive diagnosis and determine the appropriate therapeutic strategy, a CT-guided biopsy was performed (Figure 5).

**Figure 6:**
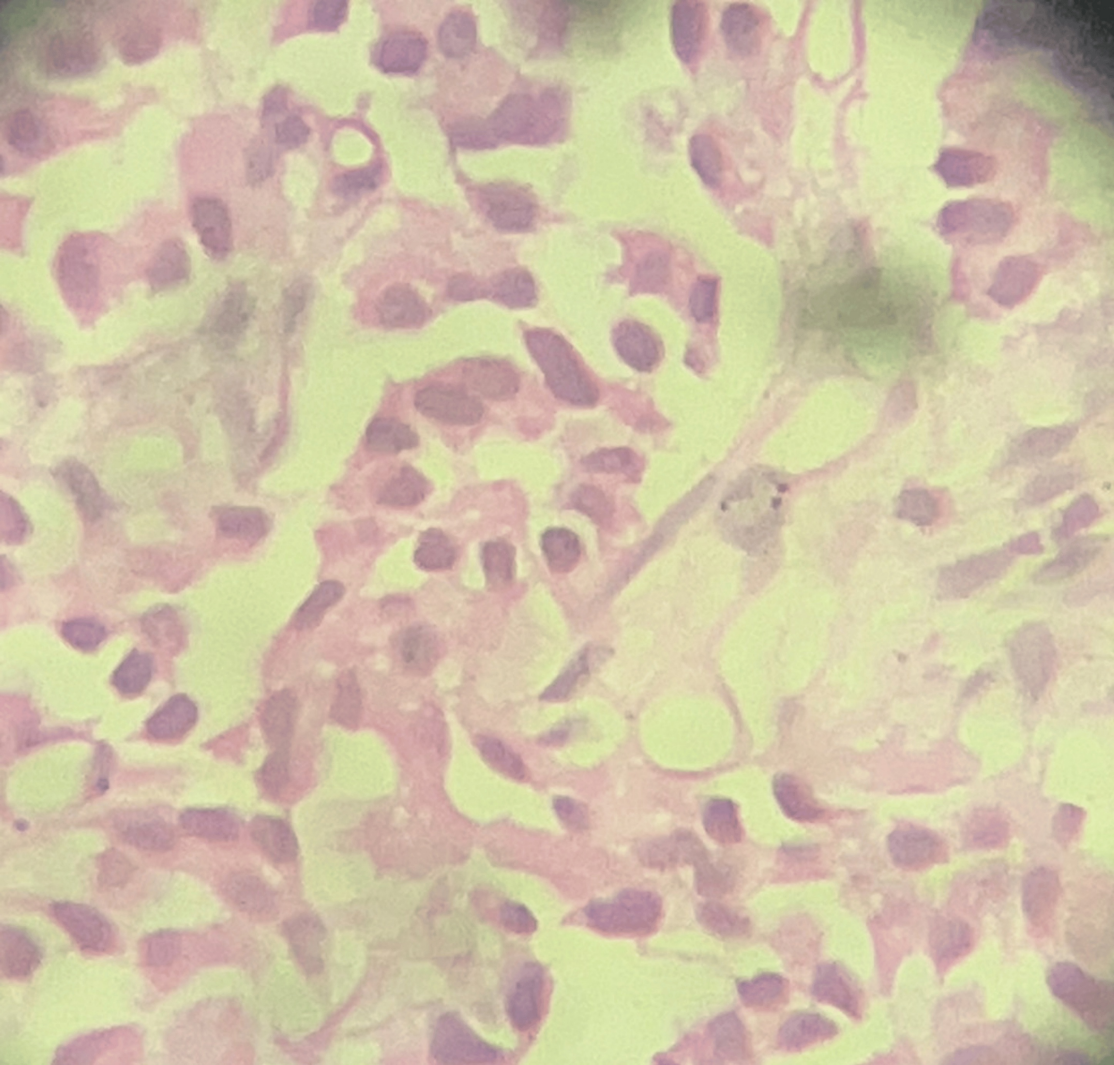
HPE (histopathological examination) of the acquired sample high power reveals fibro-collagenous tissue with neoplasm composed of nearly monomorphic cells having prominent round to oval nuclei and scanty eosinophilic cytoplasm, arranging in cords and trabeculae. Further immunohistochemistry (IHC) revealed positive staining for Synaptophysin and Chromogranin A, and a low Ki67 index, suggestive of a neuroendocrine neoplasm.

Histopathological examination (HPE) of the biopsy specimens revealed the presence of a NET, specifically a Grade 1 typical carcinoid subtype (Figure 6).

The histopathological diagnosis was further corroborated by immunohistochemical (IHC) analysis, which showed heightened expressions of Synaptophysin and CgA, both markers indicative of neuroendocrine differentiation.

The integration of radiological findings with histopathological analysis provided critical insights into the nature of the lesion. Despite the rarity of the condition, the diagnosis of a typical carcinoid tumor was unequivocally established. However, the review raises several concerns that need to be addressed for a more comprehensive evaluation. First, a functional nuclear medicine evaluation, such as a 68-Gallium-DOTATATE or 68-Gallium-DOTANOC PET scan, is mandatory for the accurate diagnosis of bronchopulmonary carcinoid tumors and should be included. Additionally, functional biochemical data, including levels of CgA, neuron-specific enolase, 5-hydroxyindoleacetic acid (5-HIAA), and ACTH, should be provided, as these neuroendocrine markers are crucial for a complete diagnostic workup.

Moreover, a detailed treatment plan should be outlined, considering both surgical and non-surgical options, tailored to the individual case. Finally, a conclusion summarizing the findings and clinical implications of the case would enhance the paper's clinical relevance. This case underscores the importance of considering uncommon entities in the differential diagnosis of pulmonary lesions and highlights the need for a thorough and multidisciplinary approach in the evaluation of such tumors.

In summary, the integration of radiological and pathological data in the case of a 39-year-old woman with a typical carcinoid tumor of the lung illustrates the value of a multidisciplinary strategy. It emphasizes the need for thorough and methodical evaluation in the diagnosis and management of rare pulmonary lesions. This case exemplifies the critical role of collaborative efforts in achieving accurate diagnoses and optimal therapeutic outcomes, thereby advancing patient care standards within the medical field.

## Discussion

This case presents a unique manifestation of a typical carcinoid tumor in a 39-year-old female who exhibited high-grade intermittent fever, chills, rigors, and breathlessness. The diagnostic process highlighted several challenges and underscored the importance of a comprehensive and multidisciplinary approach in such unusual clinical scenarios.

Pulmonary carcinoid tumors, particularly the typical variant, are known for their indolent nature and favorable prognosis [1]. However, the presentation of a high-grade intermittent fever as seen in this case is atypical, making initial diagnosis challenging. Typical carcinoids usually present with localized symptoms such as cough, hemoptysis, or recurrent infections [2]. Systemic manifestations, including fever, are rare and often lead to a broad differential diagnosis, complicating the diagnostic pathway. As previously mentioned, the clinical manifestations can vary significantly depending on the hormonal production by the tumor. For instance, tumors producing ACTH may lead to ectopic Cushing's syndrome, presenting with symptoms such as central obesity, hypertension, and glucose intolerance. This variability in clinical presentation underscores the importance of considering the tumor's endocrine activity in the diagnostic process [3]. In this case, initial clinical evaluations and imaging suggested a malignant neoplasm, highlighting the importance of considering a wide range of potential diagnoses when encountering unusual symptoms.

Radiological findings played a pivotal role in the diagnostic process. The homogeneously enhancing lesion observed on the CT scan raised suspicion of malignancy, prompting further investigation through a CT-guided biopsy [4]. This imaging characteristic, although suggestive, is not definitive for carcinoid tumors and could indicate other conditions such as primary lung cancers, metastatic lesions, or benign entities like pulmonary hamartomas [5]. The use of advanced imaging techniques is crucial in narrowing down differential diagnoses but must be corroborated with histopathological findings for definitive diagnosis.

Histopathological examination and immunohistochemical analysis confirmed the diagnosis of a typical carcinoid tumor. The presence of neuroendocrine differentiation markers, Synaptophysin and CgA, provided critical confirmation [6]. This case underscores the necessity of integrating radiological and pathological data to achieve an accurate diagnosis. In instances where radiological findings are ambiguous, biopsy and subsequent histological examination become essential.

The differential diagnosis for a homogeneously enhancing pulmonary mass is extensive and includes primary lung cancers, metastatic disease, pulmonary hamartomas, granulomatous diseases, and infectious etiologies. Each of these conditions presents distinct histopathological and clinical features that guide the diagnostic process [7]. For instance, adenocarcinomas and squamous cell carcinomas, common primary lung cancers, typically exhibit specific radiographic patterns and histological characteristics distinct from NETs. Similarly, infectious etiologies like fungal infections or tuberculosis require microbiological and histopathological confirmation [8].

The treatment of typical carcinoid tumors primarily involves surgical resection, which aims to achieve complete tumor removal and clear margins [9]. In this case, a left lower lobectomy was performed, followed by vigilant postoperative care and regular follow-up to monitor for recurrence. The prognosis for typical carcinoid tumors is generally favorable, especially when complete resection is achieved. However, the potential for recurrence or metastasis necessitates ongoing surveillance [10].

This case also highlights several difficulties encountered in the management of pulmonary carcinoid tumors. The atypical presentation with high-grade intermittent fever initially diverted clinical suspicion away from NETs. Overcoming such diagnostic challenges requires thorough history-taking, a high index of suspicion, and prompt histopathological evaluation [11]. Radiological findings, while suggestive, often overlap with other conditions, necessitating tissue biopsy for definitive diagnosis.

Another significant challenge is the histopathological differentiation of typical carcinoid tumors from other neuroendocrine neoplasms and metastatic lesions. Expert pathological evaluation, including detailed histological and immunohistochemical analysis, is crucial. Collaborative efforts between pulmonologists, thoracic surgeons, radiologists, and pathologists are essential to ensure comprehensive evaluation and accurate diagnosis [12]. Regular multidisciplinary meetings can facilitate coordinated care and improve patient outcomes.

This case underscores the importance of a multidisciplinary approach in managing atypical clinical presentations and radiographic findings. The combination of clinical acumen, radiological scrutiny, and histopathological analysis forms the cornerstone of precise diagnostic processes and tailored therapeutic strategies [13]. Such a comprehensive approach ensures optimal patient outcomes by addressing diagnostic ambiguities and therapeutic uncertainties.

Drawing from the existing literature, this case contributes to the broader understanding of pulmonary carcinoid tumors and emphasizes the importance of considering these neoplasms in differential diagnoses of pulmonary lesions and systemic symptoms [14-16]. The integration of clinical, radiological, and pathological data is crucial in navigating the complexities of diagnostic and therapeutic decision-making, ensuring that patients receive personalized and effective care.

## Conclusions

The case of a 39-year-old woman with a typical carcinoid tumor of the lung underscores the importance of a multidisciplinary approach in achieving precise diagnoses and optimal therapeutic outcomes. It demonstrates the crucial role of collaborative efforts in enhancing patient care and emphasizes the need for thorough and systematic evaluation in managing rare pulmonary lesions. By integrating empirical evidence and scholarly analysis, healthcare professionals can effectively navigate the complexities of diagnostic and therapeutic decision-making, thereby advancing the standards of patient care.

## References

[1] (2015). WHO Classification of Tumours of the Lung, Pleura, Thymus and Heart. World Health Organization. https://publications.iarc.who.int/Book-And-Report-Series/Who-Classification-Of-Tumours/WHO-Classification-Of-Tumours-Of-The-Lung-Pleura-Thymus-And-Heart-2015.

[2] Gustafsson BI, Kidd M, Chan A, Malfertheiner MV, Modlin IM (2008). Bronchopulmonary neuroendocrine tumors. Cancer.

[3] Garg R, Kumar R, Singh P, Kshetrimayum S (2019). Atypical carcinoid tumor of the lung: a rare entity. Lung India.

[4] Hartman TE, Primack SL, Lee KS, Swensen SJ, Müller NL (1994). CT of bronchial and bronchiolar diseases. Radiographics.

[5] Pelosi G, Sonzogni A, Harari S (2010). Pulmonary neuroendocrine tumors: an update. Diagnostic Histopathol.

[6] Gould VE, Wiedenmann B, Lee I (1987). Synaptophysin expression in neuroendocrine neoplasms as determined by immunocytochemistry. Am J Pathol.

[7] Pelosi G, Bianchi F, Dama E (2018). Most high-grade neuroendocrine tumours of the lung are likely to secondarily develop from pre-existing carcinoids: innovative findings skipping the current pathogenesis paradigm. Virchows Arch.

[8] Yamaguchi M, Hirai F, Taguchi K (2017). A typical carcinoid tumor of the lung presenting with pure persistent ground-glass opacity on high-resolution computed tomography: a case report. Surg Case Rep.

[9] Windmöller BA, Greiner JF, Förster C (2019). A typical carcinoid of the lung - a case report with pathological correlation and propagation of the cancer stem cell line BKZ1 with synaptophysin expression. Medicine (Baltimore).

[10] Caplin ME, Baudin E, Ferolla P (2015). Pulmonary neuroendocrine (carcinoid) tumors: European Neuroendocrine Tumor Society expert consensus and recommendations for best practice for typical and atypical pulmonary carcinoids. Ann Oncol.

[11] Fink G, Krelbaum T, Yellin A, Bendayan D, Saute M, Glazer M, Kramer MR (2001). Pulmonary carcinoid: presentation, diagnosis, and outcome in 142 cases in Israel and review of 640 cases from the literature. Chest.

[12] Filosso PL, Guerrera F, Evangelista A (2016). Management of bronchial carcinoids according to the latest WHO classification of pulmonary neuroendocrine tumors. Exp Rev Resp Med.

[13] Öberg K, Hellman P, Ferolla P, Papotti M (2012). Neuroendocrine bronchial and thymic tumors: ESMO Clinical Practice Guidelines for diagnosis, treatment and follow-up. Ann Oncol.

[14] Maroun J, Kocha W, Kvols L (2006). Guidelines for the diagnosis and management of carcinoid tumours. Part 1: The gastrointestinal tract. A statement from a Canadian National Carcinoid Expert Group. Curr Oncol.

[15] Travis WD, Brambilla E, Noguchi M (2011). International association for the study of lung cancer/american thoracic society/european respiratory society international multidisciplinary classification of lung adenocarcinoma. J Thorac Oncol.

[16] Inamura K (2018). Update on immunohistochemistry for the diagnosis of lung cancer. Cancers (Basel).

